# Psychotropic Use Among Classroom Teachers in Espírito Santo: A Cross‐Sectional Study

**DOI:** 10.1002/pds.70327

**Published:** 2026-01-29

**Authors:** Yohan Cancilheri Mazzini, Paulo Vitor Ramos Vitori, Kimberly Domingos Schneider, Gustavo Magno Baldin Tiguman, Kérilin Stancine Santos Rocha, Dyego Carlos Souza Anacleto de Araújo

**Affiliations:** ^1^ Graduate Program in Pharmaceutical Sciences, Laboratory for Innovation in Health Care Federal University of Espírito Santo Vitoria Brazil; ^2^ Laboratory for Innovation in Health Care, Department of Pharmaceutical Sciences Federal University of Espírito Santo Vitoria Brazil; ^3^ Faculty of Pharmaceutical Sciences University of São Paulo São Paulo Brazil

**Keywords:** antidepressants, Brazil, cross‐sectional studies, mental health, pharmacoepidemiology, psychotropic drugs, school teachers

## Abstract

**Purpose:**

To assess the prevalence of psychotropic use among classroom teachers and to identify associated factors.

**Methods:**

A cross‐sectional study was conducted between January and February 2024 in Espírito Santo, Brazil. The study included teachers from 20 state schools selected through probabilistic sampling. Data were collected in person using a self‐administered questionnaire that addressed sociodemographic characteristics, working conditions, mental health history, mental health screening scales, and the use of psychotropic medications. Poisson regression with robust variance was employed to estimate the prevalence ratio of psychotropic and antidepressant use. The study was approved by the research ethics committee.

**Results:**

The study included 453 teachers. The prevalence of psychotropic medication use was 20.0% (95% CI: 16.9%–22.9%), while the prevalence of antidepressant use was 16.9% (95% CI: 13.8%–19.8%). In the multivariate analysis, a higher prevalence of psychotropic use was observed among cisgender women (PR = 1.91; 95% CI: 1.07–3.39) and teachers with depressive symptoms (PR = 2.30; 95% CI: 1.26–4.22). Antidepressant use was also more frequent among cisgender women (PR = 2.07; 95% CI: 1.12–3.85) and those with depressive symptoms (PR = 2.01; 95% CI: 1.05–3.82), while teachers working in schools located in Santa Teresa showed a lower prevalence compared to those in Vitória (PR = 0.31; 95% CI: 0.10–0.90).

**Conclusion:**

The findings indicate a considerable prevalence of psychotropic and antidepressant use among teachers, particularly among cisgender women and those presenting depressive symptoms.

## Key Points

- This study provides novel evidence on the prevalence of psychotropic medication use among classroom teachers in Brazil, a population underrepresented in international literature.
- One in five teachers reported psychotropic medication use, highlighting a substantial mental health treatment burden in this occupational group.
- Psychotropic and antidepressant use was significantly higher among cisgender women compared to cisgender men, and among teachers with depressive symptoms compared to those without such symptoms.
- Teachers working in rural municipalities, such as Santa Teresa, reported lower antidepressant use compared to those in the state capital, suggesting contextual disparities in access or prescription patterns.

## Plain Language Summary

This study looked at how many school teachers use medications for mental health, such as antidepressants and other psychotropic drugs, and what factors are linked to this use. Between January and February 2024, we surveyed 453 teachers from 20 public schools in Espírito Santo, Brazil. Teachers answered questions about their personal and work characteristics, mental health, and use of medications. We found that one in five teachers reported using a psychotropic medication, and about one in six used antidepressants. Women and teachers who showed symptoms of depression were more likely to use these medications. Teachers working in smaller towns, such as Santa Teresa, were less likely to use antidepressants than those in the state capital, Vitória. These findings show that mental health problems and the use of related medications are common among teachers. The results highlight the need for better support for teachers' mental health and fair access to care across different regions.

## 1 Introduction

Teachers are often considered a high‐risk group for developing mental disorders due to the demands and pressures of their profession, which include fostering student development and formation. These responsibilities significantly increase the emotional and psychological burden of teaching [1, 2, 3]. The pressure to achieve academic results, limited resources, and escalating administrative demands contribute to a stressful work environment, thereby elevating the risk of mental disorders, including anxiety, depression, and professional burnout. Such challenges affect not only teachers' well‐being but also the quality of education provided [3, 4, 5].

A systematic review by Agyapong et al. [6] reported a high prevalence of anxiety, depression, burnout, and stress among teachers. Anxiety symptoms were observed in 38% to 41.2% of teachers, while depression symptoms ranged from 4% to 77%. Stress symptoms varied between 8.3% and 87.1%, and burnout symptoms ranged from 25.12% to 74%. These findings underscore a strong link between the teaching profession and an elevated risk of developing mental disorders. The Covid‐19 pandemic further intensified these challenges, introducing substantial pressures that adversely affected teachers' mental, social, psychological, educational, and economic well‐being. As a result, teachers experienced unprecedented levels of stress and fear, leading to a marked decline in their quality of life and mental health [7, 8]. This deterioration in teachers' mental health has important clinical and public health implications. One of the most evident manifestations of this issue is the growing use of psychotropic medications among educators.

In Brazil, the prevalence of psychotropic medication use in the general population has been extensively studied, highlighting growing concerns about mental health. Research indicates that between 2.6% and 11.7% of Brazilians use these medications, with antidepressants being the most frequently consumed [9, 10, 11, 12, 13]. Despite these national estimates, evidence on psychotropic medication use among specific occupational groups, particularly teachers, remains limited. This is especially relevant considering the high prevalence of mental health symptoms reported in this professional category. Studies by Coledam and Silva [14] and Silva et al. [15], conducted in schools in Londrina and Minas Gerais, reported prevalence rates of psychotropic medication use of 27.7% and 30.4%, respectively. However, these studies did not aim to comprehensively examine the use of these medications. Additionally, other studies relied on small, non‐probabilistic samples, further limiting generalizability [16, 17].

The lack of specific research on psychotropic medication uses among teachers working in high‐demand environments and the prevalence of associated mental disorders highlights a significant gap in the scientific literature [15, 18]. Therefore, this study aims to address this gap by investigating the prevalence of psychotropic medication use and associated factors among teachers from a regional education authority in Espírito Santo, Brazil.

## 2 Methods

### 2.1 Study Design

This study is part of the research project “Teacher's Mental Health”. It is an analytical, cross‐sectional epidemiological investigation aimed at estimating the prevalence of psychotropic medication use among teachers in the state school system of Espírito Santo, Brazil. The reporting of this study adheres to the STROBE guidelines (Strengthening the Reporting of Observational Studies in Epidemiology) [19].

### 2.2 Study Setting

The study was conducted in state schools in Espírito Santo, Brazil, within the area served by the Carapina Regional Education Authority, which covers the municipalities of Vitória, Serra, Santa Teresa, and Fundão. Vitória, the capital of Espírito Santo, had a population of 322 869 inhabitants and is considered one of the state's main urban and administrative hubs. Serra, with 520 653 residents, is the most populous municipality in Espírito Santo and stands out as an important industrial and commercial center in the metropolitan area. Santa Teresa, in contrast, is a smaller town with 22 808 inhabitants, known for its agriculture‐based economy and for cultural and ecotourism. Fundão, with 18 014 residents, is another small municipality in the Greater Vitória area, also characterized by agricultural activity (IBGE, 2022). According to the 2023 Education Panel of the State Court of Accounts, this authority managed 65 schools and 2662 teachers.

### 2.3 Study Participants

Classroom teachers working in state schools under the jurisdiction of the Carapina Regional Education Authority (Espírito Santo, Brazil) were eligible for inclusion. To maintain sample consistency, educators in exclusively administrative positions or those on leave at the time of data collection were excluded.

Schools were selected using probability proportional to size, with the number of teachers serving as the reference for proportionality [20]. The estimated sample size was 414 teachers, calculated based on a finite population (*N* = 2662), a 95% confidence level, a 5% margin of error, an estimated outcome proportion of 27.7% [14], and a design effect (deff) of 1.5 to account for cluster sampling. Within each selected school, all eligible teachers present at the time of data collection were invited to participate, with no individual random sampling of teachers. Twenty schools were selected, distributed across the cities of Serra (*n* = 11), Vitória (*n* = 4), Santa Teresa (*n* = 3), and Fundão (*n* = 2).

### 2.4 Data Collection

Data collection occurred from January 31 to February 2, 2024, during the Pedagogical Planning Journey, using a self‐administered paper‐and‐pencil survey. The Pedagogical Planning Journey is an educational event that brings together teachers, coordinators, and school managers to reflect on pedagogical practices, review and plan curricular activities, and establish teaching strategies for the upcoming school year [21]. During the event, the researchers presented the project to raise awareness among participants, invite their participation, and distribute the research questionnaire along with the Informed Consent Form (ICF). Teachers retained the research materials and returned the complete questionnaires on the final day of the Pedagogical Planning Journey.

### 2.5 Study Variables

The dependent variables in this study were the use of psychotropic medications and, more specifically, the use of antidepressants. Information was collected on medications that participants were actively using at the time of data collection, including the active pharmaceutical ingredient, prescription status (with or without medical prescription), prescriber's specialty, and treatment start date. Medications were classified according to the “Anatomical Therapeutic Chemical Classification System” (ATC) [22]. Psychotropic medications were identified as those within the following ATC codes: N02BF (gabapentinoid analgesics), N03 (antiepileptics), N04 (anti‐Parkinson drugs), N05 (psycholeptics), and N06 (psychoanaleptics), including N06A (antidepressants). The most used subclasses of antidepressants were classified as N06AA (non‐selective monoamine reuptake inhibitors), N06AB (selective serotonin reuptake inhibitors [SSRIs]), and N06AX (other antidepressants).

Independent variables collected included: gender identity (cisgender man, cisgender woman, gender minorities [including transgender and non‐binary individuals]), sexual orientation (heterosexual, homosexual, bisexual, pansexual/asexual), age group (18–24, 25–34, 35–44, 45–59, ≥ 60 years), race/ethnicity (White, Brown, Black, Asian/Indigenous), household income (≤ 3 minimum wages; 3–5 minimum wages; > 5 minimum wages), marital status (with or without a partner), number of children (none, one, two, > two), highest academic degree (undergraduate, specialization, master's/PhD), employment type (permanent, temporary), school location (Vitória, Serra, Fundão, Santa Teresa), job satisfaction (dissatisfied, neutral, satisfied), second occupation (yes, no), teaching experience (≤ 5, 6–10, 11–15, 16–20, > 20 years), weekly workload (≤ 25, 26–40, 41–50, > 50 h), work shift (day and/or night), health insurance coverage (yes, no), insomnia symptoms (yes, no), anxiety symptoms (yes, no), depressive symptoms, and burnout symptoms (yes, no).

Anxiety symptoms were assessed using the Brazilian version of the Generalized Anxiety Disorder‐7 (GAD‐7), a seven‐item scale scored from 0 to 3, reflecting the frequency of anxiety symptoms over the past 15 days. A cutoff score of ≥ 10 was adopted to indicate the presence of anxiety symptoms [23, 24]. The GAD‐7 has demonstrated validity and reliability for the Brazilian population. Depressive symptoms were assessed using the Brazilian version of the Patient Health Questionnaire‐9 (PHQ‐9), a nine‐item scale scored from 0 to 3, assessing the frequency of depressive symptoms over the past 15 days. A cutoff score of ≥ 10 was adopted to indicate the presence of depressive symptoms. The PHQ‐9 has also demonstrated validity and reliability for the Brazilian population [25, 26].

The presence of insomnia symptoms was measured using the Brazilian version of the insomnia severity index (ISI), a two‐item instrument with five response options and a maximum score of 10. A score ≥ 6 indicated the presence of clinical insomnia symptoms [27, 28, 29]. Emotional exhaustion was assessed using the Brazilian version of the Oldenburg Burnout Inventory (OLBI), specifically the exhaustion domain, which comprises six items rated on a 4‐point Likert scale ranging from 1 (*strongly disagree*) to 4 (*strongly agree*). The cutoff point for the presence of burnout symptoms was defined as the mean value of the domain plus one standard deviation [30, 31]. The OLBI has demonstrated evidence of validity and reliability for the Brazilian population [32].

### 2.6 Bias

The data collection instrument was pre‐tested with a group of 10 teachers from a school not included in the final sample to assess clarity, comprehension, and time required for completion. Minor wording adjustments were made based on participant feedback to ensure the instrument's adequacy. To avoid the influence of recall bias, we gathered information on the current use of psychotropic medications (at the moment of data collection).

### 2.7 Data Analysis

The collected data was tabulated, and descriptive statistics were applied. Absolute and relative frequencies were calculated for categorical variables, while means and standard deviations were used for continuous variables. Prevalence ratios (PR) for the use of psychotropic and antidepressant medications were estimated alongside their respective 95% confidence intervals (CI) across various independent variables. To determine the PRs for predictor variables, Poisson regression with robust variance was employed. This method was chosen for its suitability in handling non‐normally distributed data, offering greater accuracy compared to standard approaches [33]. Variables with a *p* value < 0.20 in unadjusted analyses were included in a multivariable‐adjusted model to account for potential confounders. All statistical analyses were performed using the Statistical Package for the Social Sciences (SPSS), version 27.0.

## 3 Ethical Aspects

The research project was approved by the Research Ethics Committee involving Human Subjects at the Health Sciences Center of the Federal University of Espírito Santo (CEP/CCS/UFES), under approval number CAAE: 70203023.4.0000.5060. Participants were fully informed about the study's objectives and the voluntary nature of their involvement. Each participant provided written consent by signing the ICF, in compliance with CNS Resolution No. 466/12 (Brazil, 2012). To ensure ethical rigor, participant confidentiality and anonymity were strictly maintained throughout the study. Additionally, authorization to conduct research in schools was obtained from the State Department of Education, safeguarding alignment with institutional protocols and policies.

## 4 Results

### 4.1 Sociodemographic Profile of the Participants

Of the 525 participants who initially completed the paper‐and‐pencil survey and provided written informed consent, 72 individuals were excluded based on predefined criteria. Exclusions were due to professional roles that did not align with the study's target population of classroom teachers: directors (*n* = 6), specialized educational support teachers (*n* = 24), pedagogical coordinators (*n* = 15), and pedagogues (*n* = 21). Additionally, six participants were excluded because they did not provide a response regarding psychotropic medication use, the primary outcome of this study. After applying these criteria, the final sample included 453 participants who met the inclusion criteria.

Most participants were cisgender women (*n* = 286; 65.3%), heterosexual (*n* = 395; 87.2%), and white (*n* = 211; 46%). Most held at least one postgraduate specialization degree (*n* = 299; 66.0%). Regarding marital status, 47.5% were married (*n* = 215), and 55.4% reported having children (*n* = 251). The most common household income was between 3 and 5 minimum wages (*n* = 229; 50.6%). Additional sociodemographic information is presented in Table 1.

**TABLE 1:** Sociodemographic characteristics of teachers in Espírito Santo (*n* = 453).

Variable	*n*	%
Gender identity		
Cisgender man	146	32.2
Cisgender woman	286	63.2
Gender minorities	6	1.3
No response	15	3.3
Sexual orientation		
Heterosexual	395	87.2
Homosexual	29	6.4
Bisexual	16	3.5
Pan/Asexual	8	1.8
No response	5	1.1
Age group		
18–24 years	6	1.3
25–34 years	112	24.7
35–44 years	152	33.5
45–59 years	148	32.7
≥ 60 years	22	4.9
No response	13	2.9
Race/ethnicity		
White	211	46.6
Brown	160	35.3
Black	72	15.9
Others	6	1.3
Don't know/Prefer not to answer	3	0.7
No response	1	0.2
Household income		
≤ 3 minimum wages	72	15.9
3–5 minimum wages	229	50.6
> 5 minimum wages	148	32.6
No response	4	0.9
Marital status		
Without partner	201	44.4
With partner	251	55.4
No response	1	0.2
Number of children		
None	199	43.9
One	126	27.8
Two	93	20.5
> Two	32	7.1
No response	3	0.7
Highest academic degree		
Undergraduate	44	9.7
Specialization	299	66.0
Master's/PhD	109	24.1
No response	1	0.2
Employment type		
Permanent	159	35.1
Temporary	290	64.0
No response	4	0.9
School location		
Vitória	109	24.1
Serra	259	57.2
Fundão	26	5.7
Santa Teresa	59	13.0
Job satisfaction		
Dissatisfied	84	18.5
Neutral	43	9.5
Satisfied	322	71.1
No response	4	0.9
Second job		
Yes	105	23.2
No	339	74.8
No response	9	2.0
Teaching experience		
≤ 5 years	102	22.5
6–10 years	101	22.3
11–15 years	99	21.9
16–20 years	53	11.7
> 20 years	93	20.5
No response	5	1.1
Weekly workload		
≤ 25 h	103	22.7
26–40 h	167	36.9
41–50 h	133	29.3
> 50 h	23	5.1
No response	27	6.0
Work shift		
Day	331	73.1
Night	121	26.7
No response	1	0.2
Health insurance coverage		
No	243	53.6
Yes	210	46.4
Insomnia symptoms		
No	349	77.0
Yes	97	21.4
No response	7	1.6
Anxiety symptoms		
No	304	67.1
Yes	148	32.7
No response	1	0.2
Depressive symptoms		
No	293	64.7
Yes	159	35.1
No response	1	0.2
Burnout symptoms		
No	354	78.1
Yes	99	21.9

In terms of professional characteristics, most teachers held temporary employment contracts (*n* = 290; 64.0%). Teachers with 11 to 20 years of experience represented the largest proportion of the sample (*n* = 152; 33.7%). A significant portion reported a weekly workload of over 40 h (*n* = 156; 34.4%), with teaching being the sole professional activity for most respondents (*n* = 339; 74.8%).

### 4.2 Profile of Psychotropic Medication Use

The prevalence of psychotropic medication use among teachers was 20.0% (95% CI: 16.9%–22.9%). The number of psychotropic medications used ranged from 1 to 6 (mean = 1.92; SD = 1.08). Among those using psychotropic medications, 44.4% (*n* = 40) used one medication, 32.2% (*n* = 29) used two, and 23.4% used three or more. Antidepressant use was prevalent in 16.9% (95% CI: 13.8%–19.8%) of all teachers.

A total of 168 psychotropic medications were reported, encompassing 38 unique active pharmaceutical ingredients. The classification of these ingredients according to the Anatomical Therapeutic Chemical (ATC) system is detailed in Table 2. Antidepressants were the most used class of psychotropics, with selective SSRIs used by 9.2% (*n* = 42) of teachers and other antidepressants by 9.0% (*n* = 41). Benzodiazepine‐derived antiepileptics were used by 4.4% (*n* = 20), and benzodiazepine‐derived anxiolytics by 2.9% (*n* = 13).

**TABLE 2:** Prevalence and classes of drugs used by teachers in the state education system among the general sample of teachers (*n* = 453) and among teachers who use drugs (*n* = 90).

	*n*	%_(g)_	%_(m)_
N02BF gabapentinoid analgesics	1	0.2	1.1
N03AE antiepileptics benzodiazepine derivatives	20	4.4	22.2
N03AG antiepileptics derived from fatty acids	1	0.2	1.1
N03AX ather antiepileptics	9	2.0	10.0
N05AA phenothiazine antipsychotics with aliphatic side chain	1	0.2	1.1
N05AE indolic‐derived antipsychotics	1	0.2	1.1
N05AH antipsychotics diazepines. oxazepines. thiazepines and oxepines	8	1.8	8.9
N05AL antipsychotic benzamides	1	0.2	1.1
N05AN lithium Antipsychotics	4	0.9	4.4
N05AX other antipsychotics	1	0.2	1.1
N05BA anxiolytic benzodiazepine derivatives	13	2.9	14.4
N05BE anxiolytics derived from azaspirodecanedione	3	0.7	3.3
N05CF benzodiazepine‐related hypnotics and sedatives	7	1.5	7.8
N05CH hypnotics and sedatives melatonin receptor agonists	1	0.2	1.1
N06AA nonselective monoamine reuptake inhibitor antidepressants	11	2.4	12.2
N06AB selective serotonin reuptake inhibitor antidepressants	42	9.3	46.7
N06AX other antidepressants	41	9.0	45.6
N06BA central sympathomimetic psychostimulants	3	0.7	3.3

*Note: n* = number of drugs used; %(g) = prevalence of use in relation to the general sample (*n* = 453); %(m) = prevalence in relation to teachers who use any drug (*n* = 90).

The most frequently used active pharmaceutical ingredients were clonazepam (*n* = 20; 4.4%), escitalopram (*n* = 15; 3.3%), bupropion (*n* = 15; 3.3%), and sertraline (*n* = 14; 3.1%). The prevalence of medication used by active pharmaceutical ingredients is available in Supporting Information.

### 4.3 Medication Initiation and Prescription Patterns

Most medications in use were initiated after 2020 (*n* = 100; 59.5%). Of all medications, 84.5% (*n* = 142) were prescribed by healthcare professionals, primarily by psychiatrists (74.6%; *n* = 106), followed by general practitioners (13.4%; *n* = 19) and other medical specialists (12.0%; *n* = 17). A noteworthy 8.9% (*n* = 15) of medications were used without a prescription, while 6.5% (*n* = 11) lacked information on their prescription status.

### 4.4 Multivariate Analysis

The multivariate analysis indicated that cisgender women showed a higher prevalence of psychotropic use compared to cisgender men (PR = 1.91; 95% CI: 1.07–3.39). Teachers with depressive symptoms (PR = 2.30; 95% CI: 1.26–4.22) also exhibited a higher prevalence of psychotropic use (Table 3). The multivariate analysis also revealed that both cisgender women (PR = 2.07; 95% CI: 1.12–3.85) and teachers presenting depressive symptoms (PR = 2.01; 95% CI: 1.05–3.82) showed a higher prevalence of antidepressant use. Additionally, teachers working in schools located in Santa Teresa exhibited a significantly lower prevalence of antidepressant use compared to those in Vitória (PR = 0.31; 95% CI: 0.10–0.90) (Table 4).

**TABLE 3:** Poisson regression with robust variance for psychotropic medication use among teachers, Carapina Regional Education Superintendence, Espírito Santo, Brazil, 2024 (*n* = 453).

Variables	Psychotropic medication use			
	Unadjusted regression		Adjusted regression	
	PR (95% CI)	*p*	PR (95% CI)	*p*
Gender identity		0.002		0.068
Cisgender man	Ref.		Ref.	
Cisgender woman	2.49 (1.43–4.33)		**1.91 (1.07–3.39)**	
Gender minorities	1.61 (0.21–12.20)		0.80 (0.10–6.42)	
Sexual orientation		0.798		
Heterosexual	Ref.			
Homosexual	0.90 (0.36–2.22)			
Bisexual	1.58 (0.64–3.89)			
Pan/Asexual	1.26 (0.31–5.12)			
Age group		0.442		
18–24 years	Ref.			
25–34 years	1.02 (0.14–7.60)			
35–44 years	1.15 (0.16–8.46)			
45–59 years	1.48 (0.20–10.79)			
≥ 60 years	0.55 (0.05–6.02)			
Race/ethnicity		0.325		
White	Ref.			
Brown	0.79 (0.50–1.25)			
Black	0.55 (0.27–1.11)			
Asian/Indigenous	0.96 (0.23–3.94)			
Household income		0.758		
≤ 3 minimum wages	Ref.			
3–5 minimum wages	1.25 (0.66–2.35)			
> 5 minimum wages	1.26 (0.65–2.48)			
Marital status		0.507		
With partner	Ref.			
Without partner	0.87 (0.58–1.31)			
Number of children		0.931		
None	Ref.			
One	1.18 (0.72–1.90)			
Two	1.02 (0.58–1.79)			
> Two	0.98 (0.41–2.31)			
Highest academic degree		0.421		
Undergraduate	Ref.			
Specialization	0.67 (0.36–1.25)			
Master's/PhD	0.81 (0.40–1.61)			
Employment type		0.020		0.164
Permanent	Ref.		Ref.	
Temporary	0.61 (0.40–0.92)		0.73 (0.47–1.14)	
School location		0.156		0.245
Vitória	Ref.		Ref.	
Serra	0.77 (0.48–1.21)		0.77 (0.47–1.25)	
Fundão	0.74 (0.29–1.92)		1.00 (0.37–2.71)	
Santa Teresa	0.39 (0.16–0.95)		0.39 (0.15–1.03)	
Job satisfaction		0.108		0.709
Dissatisfied	Ref.		Ref.	
Neutral	0.92 (0.45–1.89)		0.98 (0.46–2.10)	
Satisfied	0.61 (0.37–0.99)		1.24 (0.69–2.23)	
Second occupation		0.032		0.322
Yes	Ref.		Ref.	
No	1.64 (1.06–2.54)		1.28 (0.79–2.06)	
Teaching experience		0.467		
≤ 5 years	Ref.			
6–10 years	1.33 (0.66–2.66)			
11–15 years	1.47 (0.74–2.91)			
16–20 years	1.82 (0.86–3.88)			
> 20 years	1.72 (0.88–3.37)			
Weekly workload		0.990		
≤ 25 h	Ref.			
26–40 h	0.98 (0.56–1.72)			
41–50 h	1.04 (0.59–1.86)			
> 50 h	0.89 (0.30–2.60)			
Work shift		0.952		
Day	Ref.			
Night	0.99 (0.62–1.57)			
Health insurance coverage		0.024		0.109
No	Ref.		Ref.	
Yes	1.64 (1.06–2.53)		1.46 (0.92–2.31)	
Insomnia symptoms		0.000		0.222
No	Ref.		Ref.	
Yes	2.76 (1.81–4.21)		1.38 (0.82–2.29)	
Anxiety symptoms		0.000		0.409
No	Ref.		Ref.	
Yes	3.20 (2.09–4.88)		1.29 (0.70–2.38)	
Depressive symptoms		0.000		0.007
No	Ref.		Ref.	
Yes	3.87 (2.49–6.03)		**2.30 (1.26–4.22)**	
Burnout symptoms		< 0.01		0.204
No	Ref.		Ref.	
Yes	2.63 (1.73–399)		1.42 (0.83–2.44)	

*Note:* Multivariate regression was adjusted by the following independent variables: gender identity, employment type, school location, job satisfaction, second occupation, health insurance coverage, insomnia symptoms, anxiety symptoms, depressive symptoms, and burnout symptoms (*p* < 0.20 in the unadjusted analyses).

**TABLE 4:** Poisson regression with robust variance for antidepressant medication use among teachers, Carapina Regional Education Superintendence, Espírito Santo, Brazil, 2024 (*n* = 453).

Variables	Antidepressant medication use			
	Unadjusted regression		Adjusted regression	
	PR (95% CI)	*p*	PR (95% CI)	*p*
Gender identity		0.006		0.051
Cisgender man	Ref.		Ref.	
Cisgender woman	2.44 (1.34–4.43)		**2.07 (1.12–3.85)**	
Gender minorities	1.87 (0.25–14.31)		0.80 (0.10–6.45)	
Sexual orientation		0.836		
Heterosexual	Ref.			
Homosexual	1.05 (0.42–2.60)			
Bisexual	1.52 (0.55–4.17)			
Pan/Asexual	1.52 (0.37–6.20)			
Age group		0.728		
18–24 years	Ref.			
25–34 years	0.86 (0.11–6.46)			
35–44 years	1.03 (0.14–7.56)			
45–59 years	1.18 (0.16–8.63)			
≥ 60 years	0.55 (0.05–6.02)			
Race/ethnicity		0.135		0.234
White	Ref.		Ref.	
Brown	0.77 (0.47–1.26)		0.75 (0.44–1.25)	
Black	0.41 (0.17–0.96)		0.42 (0.18–1.01)	
Asian/Indigenous	1.09 (0.26–4.50)		0.87 (0.20–3.70)	
Household income		0.868		
≤ 3 minimum wages	Ref.			
3–5 minimum wages	1.09 (0.55–2.13)			
> 5 minimum wages	1.19 (0.59–2.41)			
Marital status		0.331		
With partner	Ref.			
Without partner	0.80 (0.51–1.25)			
Number of children		0.976		
None	Ref.			
One	1.10 (0.65–1.88)			
Two	0.97 (0.53–1.79)			
> Two	0.94 (0.37–2.41)			
Highest academic degree		0.220		
Undergraduate	Ref.			
Specialization	0.63 (0.32–1.26)			
Master's/PhD	0.93 (0.44–1.95)			
Employment type		0.007		0.104
Permanent	Ref.		Ref.	
Temporary	0.534 (0.34–0.84)		0.67 (0.42–1.09)	
School location		0.154		0.142
Vitória	Ref.		Ref.	
Serra	0.71 (0.43–1.16)		0.67 (0.40–1.14)	
Fundão	0.67 (0.23–1.93)		0.78 (0.26–2.33)	
Santa Teresa	0.37 (0.14–0.97)		**0.31 (0.10–0.90)**	
Job satisfaction		0.153		0.556
Dissatisfied	Ref.		Ref.	
Neutral	1.03 (0.48–2.21)		1.04 (0.46–2.34)	
Satisfied	0.63 (0.37–1.08)		1.38 (0.73–2.61)	
Second occupation		0.288		
Yes	Ref.			
No	1.32 (0.80–2.16)			
Teaching experience		0.231		
≤ 5 years	Ref.			
6–10 years	1.09 (0.50–2.40)			
11–15 years	1.29 (0.60–2.75)			
16–20 years	2.09 (0.95–3.74)			
> 20 years	1.82 (0.89–3.74)			
Weekly workload		0.857		
≤ 25 h	Ref.			
26–40 h	0.78 (0.43–1.42)			
41–50 h	0.94 (0.51–1.72)			
> 50 h	0.94 (0.32–2.77)			
Work shift		0.866		
Day	Ref.			
Night	1.04 (0.63–1.73)			
Health insurance coverage		0.056		0.174
No	Ref.		Ref.	
Yes	1.57 (0.98–2.51)		1.41 (0.86–2.31)	
Insomnia symptoms		0.000		0.306
No	Ref.		Ref.	
Yes	2.59 (1.64–4.12)		1.33 (0.77–2.32)	
Anxiety symptoms		0.000		0.190
No	Ref.		Ref.	
Yes	3.15 (1.99–4.99)		1.55 (0.81–2.98)	
Depressive symptoms		0.000		0.034
No	Ref.		Ref.	
Yes	3.54 (2.21–5.69)		**2.01 (1.05–3.82)**	
Burnout symptoms				
No	Ref.	< 0.01	Ref.	0.162
Yes	2.74 (1.74–4.32)		1.51 (0.85–2.70)	

*Note:* Multivariate regression was adjusted by the following independent variables: gender identity, race/ethnicity, employment type, school location, job satisfaction, second occupation, health insurance coverage, insomnia symptoms, anxiety symptoms, depressive symptoms, and burnout symptoms (*p* < 0.20 in the unadjusted analyses).

## 5 Discussion

This study provides empirical evidence on the prevalence and factors associated with psychotropic medication use among teachers, contributing to the understanding of mental health care patterns in this professional group. The prevalence of psychotropic medication use observed in this study was notably higher than that reported in studies conducted with the general adult population in Brazil, which range from 2.7% to 11.7% [9, 11, 12, 34, 35]. Similar trends have been identified in research specifically focused on teachers. A cross‐sectional study by Coledam and Silva [14] conducted with municipal schoolteachers in Londrina reported a prevalence of 27.7% for psychotropic medication use. Likewise, Tostes et al. [36] found that 32.3% of 1201 public school teachers in Paraná used psychotropic medication. These findings reinforce the hypothesis that teachers also constitute an occupational group with higher vulnerability to psychotropic medication prescription [1, 2, 3, 37].

However, the high prevalence of psychotropic medication observed among teachers in our sample warrants reflection considering the broader literature on teacher mental health. Previous studies have documented that teaching is associated with elevated occupational demands and emotional burden, which have been linked to psychological distress and mental disorders [3, 38, 39, 40]. While our data do not allow conclusions about causal pathways or the specific motivations for psychotropic use among teachers, the observed associations with depressive symptoms suggest that mental health challenges may be particularly relevant in this professional group. In this context, it is important that mental health interventions for teachers include not only clinical support but also organizational strategies, as indicated by prior research in occupational health [17]. Future longitudinal studies are needed to determine whether and how workplace conditions influence psychotropic medication use over time and to establish causal relationships.

Notably, most psychotropic medications used by teachers were initiated after 2020. This trend aligns with the broader increase in psychotropic use across the country, particularly following the Covid‐19 pandemic, a period marked by a sharp rise in the sales of antidepressant and anxiolytic medications [10, 41, 42, 43, 44, 45, 46, 47, 48]. Therefore, psychiatrists were the main professionals responsible for prescribing the medications. This finding differs from other studies in the Brazilian literature, where general practitioners are commonly identified as the primary source of psychotropic prescriptions [44, 49]. Recent evidence suggests a growing trend toward a greater role for psychiatrists in psychotropic prescribing practices [13]. This shift may reflect an expansion in the number of psychiatrists working in both public and private healthcare settings, as well as easier access to specialists among formally employed individuals covered by private health plans.

Our study found that being a cisgender woman is significantly associated with an increased prevalence of psychotropic medication use. Female teachers more frequently face work‐related challenges, unjust institutional pressures, and complicated interpersonal relationships, compounded by specific stressors such as the glass ceiling effect, gender stereotypes, and inequality in decision‐making positions [50, 51]. The overload experienced by many women, who, in addition to their teaching roles, bear domestic and family responsibilities such as childcare, household management, and caregiving for elderly relatives, contributes to a high prevalence of conditions such as anxiety, depression, and insomnia, which are associated with greater use of antidepressants and other psychotropic medications [52, 53, 54]. Additionally, the belief in the fragility of the female sex may still influence unequal healthcare treatment by physicians, leading to a higher number of mental disorder diagnoses and increased prescriptions for psychotropic medications [55].

In our study, higher psychotropic medication use was observed among teachers who screened positive for depressive symptoms. These findings are consistent with a previous cross‐sectional study conducted with Brazilian elementary school teachers, which found individuals with common mental disorders, including depression, were more likely to use medications for psychological disorders, particularly psychotropics [14]. Several mechanisms could explain these findings. First, psychotropics such as antidepressants are a standard pharmacological intervention for major depressive disorder, as evidenced by a network meta‐analysis that confirmed common antidepressants were more effective than placebo for acute treatment of this condition [56]. Second, many patients do not achieve full remission of depressive symptoms; that is, patients often relapse while on psychotropic medication. The presence of treatment‐resistant depression is a recognized clinical reality, meaning that the observed concurrent use of psychotropic medications and depressive symptoms may be a result of partial response or treatment failure [57]. Third, occupational stressors related to teaching, such as workload, emotional demands, classroom management, and administrative pressures, increase the incidence and severity of depressive symptoms, thereby indicating the need for pharmacological treatment [6].

The lower prevalence of antidepressant use observed among teachers in Santa Teresa compared with those in Vitória may be related to contextual differences between the municipalities. Santa Teresa is a smaller municipality, characterized by predominantly rural features, lower population density, and closer community ties, factors that may contribute to greater social support and a lower perception of occupational stress. In contrast, Vitória, as the state capital and an urban administrative center, presents greater professional competitiveness, heavier workloads, and a more intense work pace, which may increase the risk of psychological distress and, consequently, the likelihood of psychotropic prescription and use. Furthermore, in smaller municipalities such as Santa Teresa, the limited availability of specialized professionals may lead to underdiagnosis of these cases. Therefore, the differences observed between municipalities may reflect not only variations in the actual prevalence of mental distress but also structural inequalities in access to mental health care.

The findings of this study should be interpreted considering its contextual characteristics. The research was conducted exclusively among teachers working in public state schools in Espírito Santo, Brazil, within the jurisdiction of the Carapina Regional Education Authority, which includes urban and rural municipalities. The sample included teachers from both elementary and secondary education levels but did not encompass private or municipal schools. Therefore, the results may not be directly generalized to other educational settings, such as private institutions or different regions of the country, where working conditions, employment stability, and access to health care services may differ. Data collection was based on self‐reporting, which may introduce response biases. To minimize the possibility of memory bias, data were collected only on medications in use during the data collection period but excluded information on medications that participants had previously used and discontinued before the study began.

Misclassification bias is also possible due to the self‐reported nature of both psychotropic medication use and mental health symptoms, which may lead to under‐ or overestimation of prevalence. Importantly, the survey was anonymous and collected no identifiable personal data, which likely minimized social desirability bias and may have increased the reliability of reports of medication use and mental health symptoms. Furthermore, the healthy worker effect should be considered, as only active teachers were included in the study. Teachers who were on medical leave or had withdrawn from work due to mental health conditions, potentially those with more severe symptoms, were not represented, which may have led to an underestimation of the true prevalence of psychotropic medication use.

The authors emphasize that the use of psychotropic medications may reflect both individual mental health needs and the broader psychosocial pressures experienced in the school environment. While medication use was assessed in this study, it is essential to highlight that mental health strategies cannot rely solely on pharmacological interventions. Immediate efforts to support teachers' well‐being should include non‐pharmacological measures such as improving sleep quality, encouraging regular physical activity, and fostering supportive school environments.

## 6 Conclusion

This study identified a high prevalence of psychotropic medication use among teachers, with antidepressants being the most reported class. The observed associations, particularly with depressive symptoms, suggest that mental health concerns may be highly relevant within this professional group. Although the cross‐sectional design does not allow causal inferences or determination of the motivations for psychotropic use, these findings contribute to the understanding of mental health care patterns among teachers in Brazil.

Policies should aim at improving working conditions, reducing work‐related stressors, and increasing access to mental health resources, including psychiatric care. Furthermore, preventive strategies, such as mental health education and stress management programs, should be integrated into teacher training and professional development, fostering a supportive work environment that prioritizes mental well‐being and reduces the reliance on medication as the primary coping mechanism.

## Funding

This study was financed in part by the Coordenação de Aperfeiçoamento de Pessoal de Nível Superior—Brasil (CAPES)—Finance Code 001 and Fundação de Amparo à Pesquisa e Inovação do Estado do Espírito Santo (FAPES).

## Ethics Statement

The study was approved by the Ethics Committee for Research with Human Beings (CAAE: 70203023.4.0000.5060).

## Conflicts of Interest

The authors declare no conflicts of interest.

## Supporting information


**Data S1:** pds70327‐sup‐0001‐Supinfo.docx.

## Data Availability

De‐identified individual‐level data and the data dictionary are available from the corresponding author upon reasonable request.
