# Supplementary material for: Psychotropic Use Among Classroom Teachers in Espírito Santo: A Cross‐Sectional Study
Source: Pharmacoepidemiol Drug Saf. 2026 Jan 29;35(2):e70327. doi: 10.1002/pds.70327 (PMC12856136; doi:10.1002/pds.70327)
Supplement: Supplementary file 1 — Data S1: pds70327‐sup‐0001‐Supinfo.docx. [file PDS-35-e70327-s001.docx]

| **Prevalence and classes of drugs used by teachers in the state education system among the general sample of teachers (n=453) and among teachers who use drugs (n=90)** | | | |
| --- | --- | --- | --- |
| **Drug** | **n** | **%_(g)_** | **%_(m)_** |
| N02BF Gabapentinoid analgesics | **1** | **0.2** | **1.1** |
| Pregabalin | 1 | 0.2 | 1.1 |
| N03AE Antiepileptics benzodiazepine derivatives | **20** | **4.4** | **22.2** |
| Clonazepam | 20 | 4.4 | 22.2 |
| N03AG Antiepileptics derived from fatty acids | **1** | **0.2** | **1.1** |
| Valproex sodium | 1 | 0.2 | 1.1 |
| N03AX Other antiepileptics | **9** | **2.0** | **10.0** |
| Canabidiol | 2 | 0.4 | 2.2 |
| Lamotrigine | 4 | 0.9 | 4.4 |
| Topiramate | 3 | 0.7 | 3.3 |
| N05AA Phenothiazine antipsychotics with aliphatic side chain | **1** | **0.2** | **1.1** |
| Levomepromazine | 1 | 0.2 | 1.1 |
| N05AE Indolic-derived antipsychotics | **1** | **0.2** | **1.1** |
| Lurasidone | 1 | 0.2 | 1.1 |
| N05AH Antipsychotics diazepines. oxazepines. thiazepines. and oxepines | **8** | **1.8** | **8.9** |
| Quetiapine | 8 | 1.8 | 8.9 |
| N05AL Antipsychotic benzamides | **1** | **0.2** | **1.1** |
| Sulpiride | 1 | 0.2 | 1.1 |
| N05AN Lithium Antipsychotics | **4** | **0.9** | **4.4** |
| Lithium | 4 | 0.9 | 4.4 |
| N05AX Other antipsychotics | **1** | **0.2** | **1.1** |
| Risperidone | 1 | 0.2 | 1.1 |
| N05BA Anxiolytic Benzodiazepine Derivatives | **13** | **2.9** | **14.4** |
| Alprazolam | 7 | 1.5 | 7.8 |
| Clobazam | 1 | 0.2 | 1.1 |
| Chlordiazepoxide | 1 | 0.2 | 1.1 |
| Cloxazolam | 1 | 0.2 | 1.1 |
| Diazepam | 3 | 0.7 | 3.3 |
| N05BE Anxiolytics derived from azaspirodecanedione | **3** | **0.7** | **3.3** |
| Buspirone | 3 | 0.7 | 3.3 |
| N05CF Benzodiazepine-related hypnotics and sedatives | **7** | **1.5** | **7.8** |
| Eszopiclone | 3 | 0.7 | 3.3 |
| Zolpidem | 4 | 0.9 | 4.4 |
| N05CH Hypnotics and sedatives melatonin receptor agonists | **1** | **0.2** | **1.1** |
| Ramelteone | 1 | 0.2 | 1.1 |
| N06AA Nonselective monoamine reuptake inhibitor antidepressants | **11** | **2.4** | **12.2** |
| Amitriptiline | 9 | 2.0 | 10.0 |
| Clomipramine | 1 | 0.2 | 1.1 |
| Nortriptiline | 1 | 0.2 | 1.1 |
| N06AB Selective serotonin reuptake inhibitor antidepressants | **42** | **9.3** | **46.7** |
| Citalopram | 3 | 0.7 | 3.3 |
| Escitalopram | 15 | 3.3 | 16.7 |
| Fluoxetine | 4 | 0.9 | 4.4 |
| Fluvoxamine | 1 | 0.2 | 1.1 |
| Paroxetine | 5 | 1.1 | 5.6 |
| Sertraline | 14 | 3.1 | 15.6 |
| N06AX Other antidepressants | **41** | **9.0** | **45.6** |
| Bupropione | 15 | 3.3 | 16.7 |
| Desvenlafaxine | 6 | 1.3 | 6.7 |
| Duloxetine | 4 | 0.9 | 4.4 |
| Mirtazapine | 2 | 0.4 | 2.2 |
| Trazodone | 9 | 2.0 | 10.0 |
| Venlafaxine | 5 | 1.1 | 5.6 |
| N06BA Central sympathomimetic psychostimulants | **3** | **0.7** | **3.3** |
| Lisdexanfetamine | 1 | 0.2 | 1.1 |
| Methylphenidate | 2 | 0.4 | 2.2 |
| Legend: n = number of drugs used; %(g)= prevalence of use in relation to the general sample (n=453); %(m) = prevalence in relation to teachers who use some drug (n=90) | | | |
